# Best-worst scaling preferences among patients with well-controlled epilepsy: Pilot results

**DOI:** 10.1371/journal.pone.0282658

**Published:** 2023-03-03

**Authors:** Samuel W. Terman, Hélène E. Aschmann, David W. Hutton, James F. Burke

**Affiliations:** 1 Department of Neurology, University of Michigan, Ann Arbor, Michigan, United States of America; 2 Department of Epidemiology and Biostatistics, University of California, San Francisco, San Francisco, California, United States of America; 3 Epidemiology Biostatistics and Prevention Institute, University of Zurich, Zurich, Switzerland; 4 Department of Health Management and Policy, School of Public Health, University of Michigan, Ann Arbor, Michigan, United States of America; 5 Department of Neurology, the Ohio State University, Columbus, Ohio, United States of America; Foundation IRCCS Carlo Besta Neurological Institute: Fondazione IRCCS Istituto Neurologico Carlo Besta, ITALY

## Abstract

Epilepsy is a common, serious condition. Fortunately, seizure risk decreases with increasing seizure-free time on antiseizure medications (ASMs). Eventually, patients may consider whether to stop ASMs, which requires weighing treatment benefit versus burden. We developed a questionnaire to quantify patient preferences relevant to ASM decision-making. Respondents rated how concerning they would finding relevant items (e.g., seizure risks, side effects, cost) on a Visual Analogue Scale (VAS, 0–100) and then repeatedly chose the most and least concerning item from subsets (best-worst scaling, BWS). We pretested with neurologists, then recruited adults with epilepsy who were seizure-free at least one year. Primary outcomes were recruitment rate, and qualitative and Likert-based feedback. Secondary outcomes included VAS ratings and best-minus-worst scores. Thirty-one of 60 (52%) contacted patients completed the study. Most patients felt VAS questions were clear (28; 90%), easy to use (27; 87%), and assessed preferences well (25; 83%). Corresponding results for BWS questions were 27 (87%), 29 (97%), and 23 (77%). Physicians suggested adding a ‘warmup’ question showing a completed example and simplifying terminology. Patients suggested ways to clarify instructions. Cost, inconvenience of taking medication, and laboratory monitoring were the least concerning items. Cognitive side effects and a 50% seizure risk in the next year were the most concerning items. Twelve (39%) of patients made at least one ‘inconsistent choice’ for example ranking a higher seizure risk as lower concern compared with a lower seizure risk, though ‘inconsistent choices’ represented only 3% of all question blocks. Our recruitment rate was favorable, most patients agreed the survey was clear, and we describe areas for improvement. ‘Inconsistent’ responses may lead us to collapse seizure probability items into a single ‘seizure’ category. Evidence regarding how patients weigh benefits and harms may inform care and guideline development.

## Introduction

Fifty million people have epilepsy [1]. Fortunately, antiseizure medications (ASMs) render two-thirds of patients with epilepsy seizure-free [2] and may reduce mortality by preventing seizures [3]. However, side effects are common (ranging from 10–40%, even as high as 60–90%) [4, 5], and explain up to one-quarter of variance in quality of life after seizure remission [6]. ASMs also may exert drug-drug interactions, are costly [7], and require monitoring. Thus, clinicians and patients must balance the pros and cons of treatment [8]. Particularly for patients who become seizure-free on ASMs, clinical guidelines suggest that seizure risk may eventually drop low enough to consider ASM discontinuation rather than lifelong treatment [9]. This recommendation is supported by an average post-discontinuation relapse risk (30–40%) [10, 11] falling below the usual threshold to start an ASM of 60% [12].

Existing literature poorly informs how to weigh disparate considerations. Qualitative work found seizure risk, side effects, and driving restrictions to be among the most important factors relevant to ASM discontinuation [13]. Surveys have tabulated the most important factors relevant to ASM discontinuation such as driving, seizures, and side effects or fear of long-term negative ASM consequences [14, 15]. However, neither qualitative interviews nor tabulations inform the relative importance of any factor, which would be useful to inform guidelines and policy-making [16]. Work correlating factors such as seizures or side effects with global quality of life scales has been performed [17], but such work does not directly inform how patients weigh tradeoffs inherent to the pros and cons of treatment decisions.

However, existing preference studies have omitted numerous important items (e.g., driving restrictions related to seizures, lab monitoring), lumped disparate items together (e.g., fatigue and moodiness as a single category [18]), treated seizure risk in terms of relative rather than absolute seizure risks, did not study physician-confirmed epilepsy, and did not study patients with well-controlled epilepsy. Thus, major gaps remain which could be filled by developing a new survey instrument.

Particularly, discrete choice experiments and more recently best-worst scaling (BWS) are frequently used to quantitatively measure patient preferences [19, 20]. In BWS, health outcomes or treatment options are ranked by choosing the best (or least concerning) and the worst (or most concerning) item repeatedly among subsets [21]. A core strength is that respondents are best at identifying preference extremes from a limited set of options, and both the most and least important factors inform decision processes. BWS has been used across medical conditions to clarify patient values [19, 22–26].

Here, we describe the development and pilot testing of our novel instrument eliciting patient preferences pertaining to ASM decisions in patients who have attained a period of seizure-freedom. The primary purpose of this pilot study was to assess recruitment rates and develop our questionnaire. We obtained Visual Analogue Scales (VAS) and BWS data, to gain insight into the most and least important factors to patients whose seizures have stabilized and thus might in the future be eligible for ASM discontinuation decisions.

## Materials and methods

### Participant selection

We pretested our online survey in five neurologist volunteers. This was from colleagues including epilepsy specialists (N = 3) and non-epilepsy specialists (N = 2) at our institution for feedback and questionnaire development prior to administering the survey to patients. Neurologists were asked to respond as if they were a patient.

Then, we recruited patients to take the online survey. We screened consecutive electronic medical records for patients ≥18 years seen at the University of Michigan Epilepsy Clinic to identify those seen for a primary outpatient diagnosis of epilepsy confirmed via manual chart review by an epileptologist (SWT). We reviewed clinic notes two weeks after clinic visits occurred, to allow enough time for documentation to be complete but also a short enough timespan such that the chart would contain current information. We required that patients be seizure-free at least one year to recruit patients who might be eligible for ASM discontinuation in the future. We recognize that many clinicians may consider ASM discontinuation only after two years of seizure-freedom given previous guidelines [9, 27], and thus we could have restricted to patients seizure-free at least two years. However, we chose our more inclusive criteria given it is important to ascertain preferences earlier rather than later to guide a patient’s future expectations, including patients who were seizure-free at least one year includes the subset of patients at seizure-free at least two years, current guidelines now dispense with the arbitrary “two-year” guideline [28], and in our future work enrolling patients with both shorter and longer seizure-free periods would enable a comparison across different durations of seizure-freedom. We acknowledge that certain patients such as those with particular syndromes (e.g., juvenile myoclonic epilepsy) or etiologies (e.g., genetic) may be viewed as less favorable candidates for discontinuation due to higher relapse risk. We included all seizure-free patients in this study, though, regardless of specific syndrome or etiology, to ensure broad representation across our patient population and to acknowledge that it is important to capture preferences across a range of higher versus lower-risk patients We approached patients initially by phone. If they did not answer the first call, we followed a sequentially mixed mode contact/delivery [29–31] involving two further phone calls, then up to two personalized emails. Completers received $10-$20.

Full BWS sample sizes are typically ~150–200 subjects [19, 25]. Our goal here though was to develop and pilot the survey, and thus we required at least thirty patients given that prior work has indicated N = 30 is adequate to detect difficulties for questionnaire pretesting [32].

The word ‘pilot’ applies to this study because our goals were to estimate recruitment rates, test novel data collection procedures for use in a future larger program of epilepsy survey research and inform questionnaire development.

### Procedures involving human subjects

This study was approved by the University of Michigan Institutional Review Board, HUM00177382. Respondents provided consent to participate by selecting a radio button on the first page of the survey.

### Survey development

To quantify the relative importance of disparate considerations relevant to ASM treatment decisions, our main intended survey instrument comprised a BWS exercise. BWS asks respondents to select the most and least important health outcomes from repeated subsets of a list [33]. A core strength of this approach is that respondents are best at identifying preference extremes from a limited set of options, and both the most and least important factors contain value towards understanding decision processes. BWS has been used increasingly to clarify how patients prioritize competing treatment values in conditions ranging from Parkinson’s disease [22] to diabetes [23] to cardiovascular disease [24, 25] to organ transplantation [26] among many other applications [19]. Ultimately, BWS places preferences weights for disparate outcomes onto a single common scale for immediate comparison regarding which factors are most important to patients, and by how much.

Table 1 shows the items we used in each portion of our survey, guided by literature [13, 15, 17, 18, 34–37], in addition to study team consensus. For example, literature [15] notes the importance of driving, seizures, and specific side effects such as cognition, mood, and balance [18, 34, 38]. We chose seizure risks within the next year within plausible ranges informed by seizure risk prediction literature [39]. We initially chose a 6 month driving restriction because this applies to the state of Michigan after a seizure, and ASM cost levels of $10/month and $50/month for the physician pilot also within plausible ranges. We then created the survey (S1 Appendix for physicians, then S2 Appendix for patients) by using software to arrange Table 1‘s items into blocks (shuffled subsets of the items from which the respondent chooses the most and least concerning) each of a prespecified size (number of items per block). We used the %MktBSize SAS macro [40] to identify the number of blocks (13) and block size (4 items/block) as the number satisfying a balanced incomplete block design (BIBD). BIBD is typical for BWS experiments [21], where each item appears the same number of times across the survey (‘balanced’) to explore all items equally, when showing all items simultaneously is not practical or even necessary to efficiently compare items (‘incomplete’). We then entered these parameters into the %MktBIBD SAS macro [41] to produce the exact blocks satisfying a BIBD setup. After the physician survey, we incorporated several points of qualitative feedback and made minor modifications described in the Results below prior to continuing with patient data collection.

**Table 1:** Items.

	VAS		BWS	
Item	Physician	Patient	Physician	Patient
Seizure risk in the next year: 10%	X	X	X	X
Seizure risk in the next year: 25%	X	X	X	X
Seizure risk in the next year: 50%	X	X	X	X
Driving restriction: 3 months				X
Driving restriction: 6 months	X	X	X	X
Medication-related mood changes	X	X	X	X
Medication-related thinking/memory difficulty	X	X	X	X
Medication-related sedation	X	X	X	X
Medication-related imbalance/dizziness	X	X	X	X
$10/month ASM cost	X	X	X	X
$50/month ASM cost	X	X	X	X
$100/month ASM cost				X
Lab monitoring every 6 months	X	X	X	X
Nuisance of taking ASM	X	X	X	X

Note: Because of physician feedback, we added a 3-month driving restriction and a $100/month antiseizure medication (ASM) cost for the patient best-worst scaling (BWS) survey.

Before BWS questions, as a ‘warmup’ we also asked each patient to rate each item on a 0–100 VAS scale [25] between “Not at all concerning: 0 (perfect health)” and “Extremely concerning: 100 (death).” We incorporated VAS in addition to BWS, both to test for convergence validity between the two preference elicitation types, and also because VAS provides a high degree of resolution between items on an absolute (not just relative) scale with defined anchors for increased interpretability [42].

### Analysis and study procedures

We obtained feedback about the survey via “talk-aloud” feedback as respondents were going through the survey in real-time, in addition to post-survey 10-minute interviews [43, 44]. We requested that all participants verbalize out loud their thoughts and experience for the investigator who was present while patients proceeded through the survey, particularly stressing any confusing points regarding instructions or content and explaining their processes for deciding upon responses. We presented such data by qualitatively describing the most important themes that emerged during this oral feedback, particularly feedback that would inform how to refine our questionnaire. We also posed multiple choice questions asking respondents to rate the clarity and ease of answer VAS and BWS questions using a 1–5 Likert scale.

We analyzed BWS data using both a multinomial logit regression, and also via the distribution of best-minus-worst scores [21]. The logit model included cluster robust standard errors for each respondent-block. Each item in each respondent-block represented a row of data. The outcome variable took on -1 (least concerning item), +1 (most concerning item), or 0 (neither). Independent variables were the items themselves and thus the main result of this analysis was each item’s β coefficient for choosing an item as the most (+1) rather than the least (-1) item [25, 45]. Second, we calculated each item’s best-minus-worst score for each respondent, as the number of times the respondent chose the item as the ‘most’ concerning minus the number of times they chose the item as the ‘least’ concerning. We displayed best-minus-worst and VAS scores via boxplots. We correlated mean best-minus-worst versus VAS scores across all items with a scatterplot and Pearson and Spearman correlation coefficient. Analyses were performed using SAS version 9.4 (Cary, NC) and Stata version 16 (College Station, TX). We completed each of these analyses separately for patients and physicians. Missing responses were not included in analysis.

For both VAS and BWS exercises, we tabulated the number of ‘inconsistencies,’ which we defined as choosing a more concerning item as less concerning, or a less concerning item as more concerning. An example would be if a respondent rated a 10% seizure risk as of higher concern than a 50% seizure risk, or if a respondent rated or ranked a 3-month driving restriction as of higher concern than a 6-month driving restriction.

## Results

### Physician pilot

First, to obtain expert feedback outside of our study team prior to surveying patients, we pilot tested our survey with 5 neurologist volunteers. S1 Appendix contains the physician survey. The 13 VAS questions took a mean of 2.3 minutes (standard deviation [SD] 1.3 minutes) to complete, and 13 BWS blocks took a mean of 6.7 minutes (SD 3.8 minutes) to complete. Feedback suggested adding a ‘warmup’ question showing an example completed BWS response, using more updated terminology including ‘antiseizure medication’ rather than ‘antiepileptic drug,’ simplifying language from ‘relapse’ to ‘seizure risk,’ using shorter simpler sentences, making a larger difference between cost levels, and adding in a 3-month driving restriction (which would apply to some states other than Michigan). Physicians did not identify any important omitted attributes. Because of this feedback, before proceeding the patient data collection, we added a 3-month driving restriction to our existing 6-month driving restriction and increased the maximal ASM cost to $100/month for patient BWS exercises. However, we kept VAS questions unchanged to still enable comparison for all initial items between physician and patient responses.

### Patient pilot: Recruitment rate and population description

Then, we screened 293 patients to reach a goal of 30 completers. Of the 293, 65 were eligible (epilepsy, at least 18 years old, at least one-year seizure-free, on at least one ASM), 60 were contacted, 44 were reached, and 31 completed our survey (31/60 = 52% completion rate). We did not contact all 65 because we reached our target sample size after contacting 60. Table 2 displays patient respondent characteristics. S2 Appendix contains the patient survey.

**Table 2:** Population description. N = 31. See footnote for antiseizure medications and etiologies.

		Median (interquartile range)
**Age**		38 (28–51)
**Age at epilepsy onset**		15 (7–33)
**Years since last seizure**		2 (2–6)
		**No (%)**
**Female**		17/31 (55%)
**Race**	Non-Hispanic White	25/31 (81%)
	Non-Hispanic Black	4/31 (13%)
	Asian	2/31 (6%)
**Focal epilepsy**		21/27 (78%)^a^
**Motor seizures** ^b^		29/31 (94%)
**Impairing awareness**		30/31 (97%)
**Surrogate responder**		5/31 (16%)
**Self-limiting syndrome**		0/31 (0%)
**Currently driving**		8/16 (50%)^c^

Antiseizure medications: Lamotrigine 14, levetiracetam 12, clobazam 4, oxcarbazepine 3, carbamazepine 5, lacosamide 2, phenobarbital 2, topiramate 2, zonisamide 2, cannabidiol 1, gabapentin 1, lorazepam 1, perampanel 1, pregabalin 1, rufinamide 1, valproate 1.

Etiologies: Cryptogenic 16, traumatic brain injury 3, central nervous system tumor 2, cavernous hemangioma 2, focal cortical dysplasia 2, genetic 2, choroid plexus cyst 1, hypoxic ischemic encephalopathy 1, ischemic stroke 1, intraventricular hemorrhage (neonatal) 1.

^a^For 4 patients, it could not be determined whether generalized convulsions represented focal versus generalized epilepsy.

^b^Motor includes myoclonic, tonic, clonic, and tonic-clonic.

^c^For 15 patients, the chart did not state whether the patient was driving, hence the denominator represented only those patients for whom the chart stated whether the patient was driving.

### Patient pilot: Feedback

For the 13 VAS questions, the mean completion time was 3.8 minutes (SD 1.7 minutes). Patients responded to 400/403 VAS items (99%). Patients suggested ensuring instructions were clear that even a choice of ‘0’ (not at all concerning) required a click and to clarify that our question involved how concerning would each item be if it were true (rather than how concerned are you that each item applies to you now). Likert-based feedback from patients for the VAS questions indicated that 90% somewhat or strongly agreed that instructions were clear, 90% felt the interface was easy to use, and 83% felt the exercise ascertained their preferences related to ASMs well (Table 3).

**Table 3:** Survey feedback.

**BWS**	**Strongly agree**	**Somewhat agree**	**Neutral**	**Somewhat disagree**	**Strongly disagree**
Instructions clear	12	15	-	3	-
Interface easy to use	21	8	-	1	-
Assessed preferences pertaining to ASMs well	14	9	5	2	-
**VAS**	**Strongly agree**	**Somewhat agree**	**Neutral**	**Somewhat disagree**	**Strongly disagree**
Instructions clear	16	12	-	3	-
Interface easy to use	21	6	-	3	-
Assessed preferences pertaining to ASMs well	16	9	4	1	-

For the 13 BWS question blocks, the mean completion time was 5.9 minutes (SD 3.3 minutes). Patients marked both a least and most concerning item, i.e., a complete response, for 378/403 (94%) of blocks. Patients marked either a least or most concerning item, i.e., a partial response, for all 403 blocks. When completing the BWS questions, patients requested clarity that each block requires two and only two marks (one for the most and one for the least concerning item), noted it might be helpful to warn future respondents that the task will be repetitive on purpose, and two respondents suggested the possibility of adding ASM-related psychosis as an item. Likert-based feedback from patients for the BWS questions indicated that 90% somewhat or strongly agreed that instructions were clear, 97% felt the interface was easy to use, and 77% felt the exercise ascertained their preferences related to ASMs well (Table 3).

### Patient pilot: Survey results

**Fig 1:**
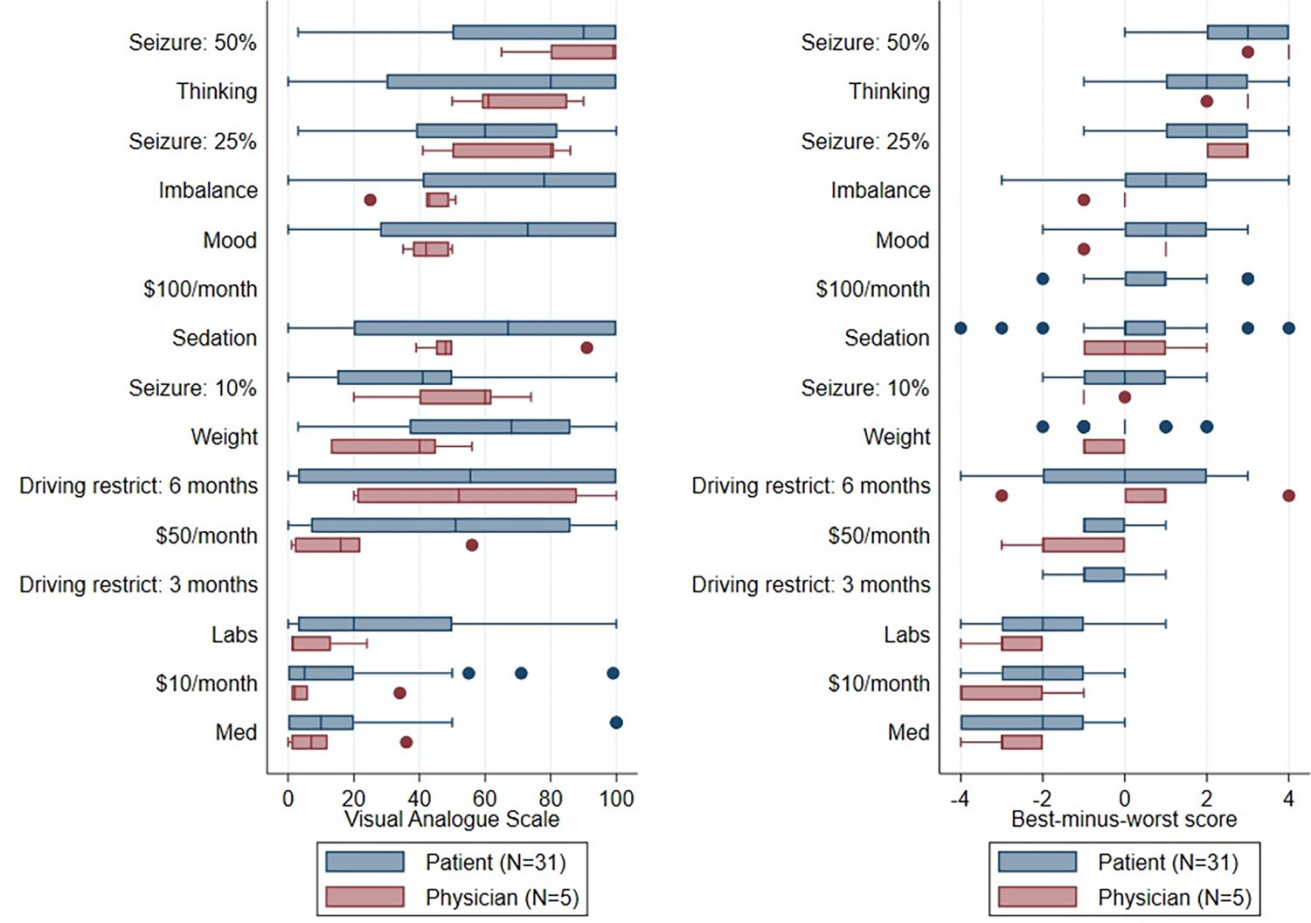
Preference distributions. Left: Visual Analogue Scale. Right: Best-minus-worst scores. Note the 3-month driving restriction and $100/month items were only asked for the patient best-worst scaling questions, in response to physician feedback.

Fig 1 displays the distributions of VAS (left) and BWS (right) responses for physicians and patients. While this pilot study was not designed to be adequately powered to compare physician and patient responses, responses followed qualitatively consistent patterns across groups. In both BWS and VAS questions, the inconvenience of taking a medication (“Med”), paying $10/month for ASMs, and having periodic lab draws for bloodwork monitoring were among the least concerning items. Medication-related thinking difficulty and a 50% chance of a seizure in the next year were among the most concerning items.

**Fig 2:**
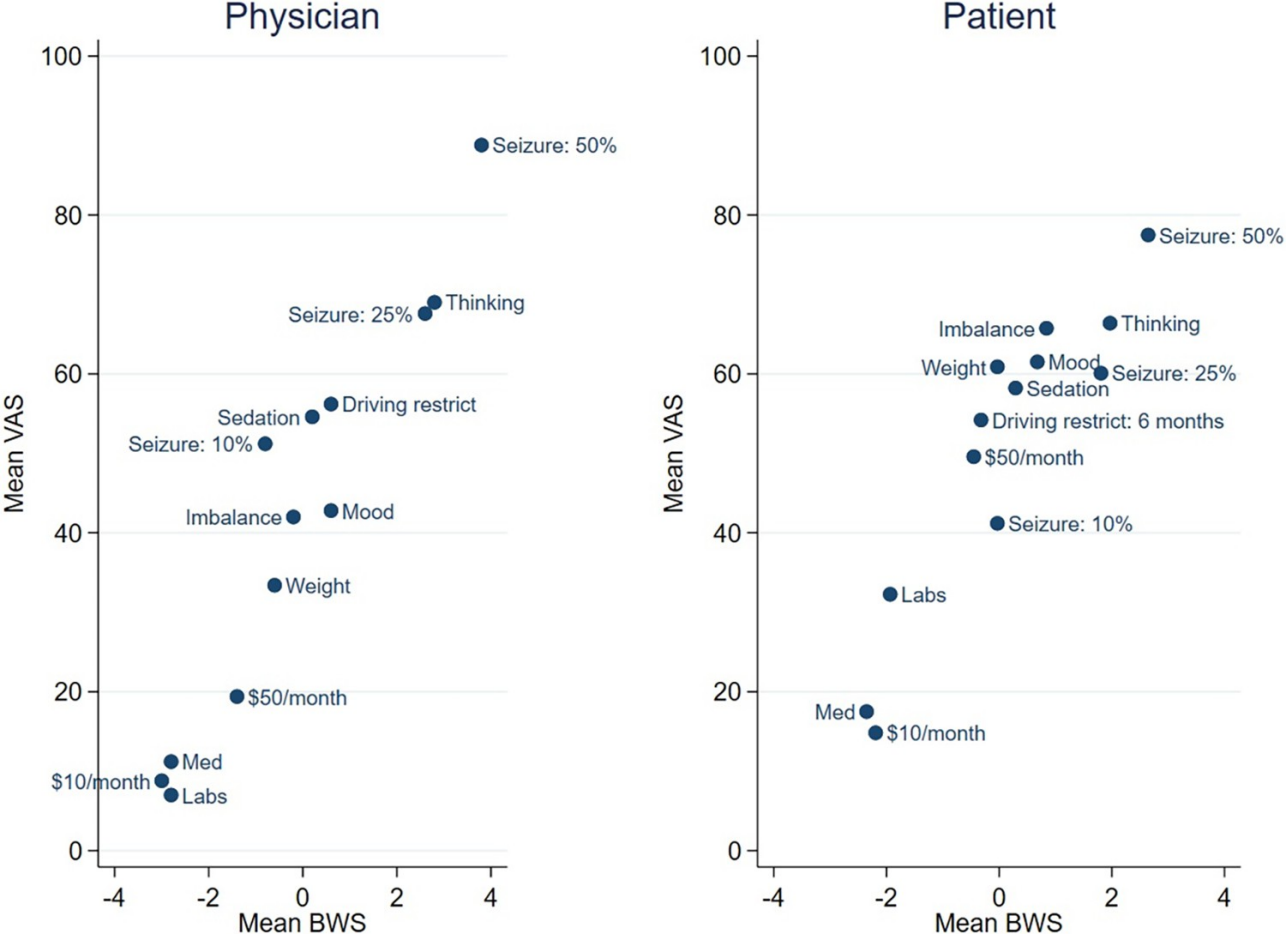
Correlation between mean VAS and BWS scores. Left: Physician responses (N = 5). Right: Patient responses (N = 31).

Fig 2 displays the correlation between the mean VAS score and the mean best-minus-worst response for each item, for physicians (left) and patients (right). Spearman and Pearson correlation coefficients were 0.94 and 0.96, respectively, comparing mean VAS versus mean best-minus-worst scores for physicians. Both measures of correlation were 0.92 for patients.

**Fig 3:**
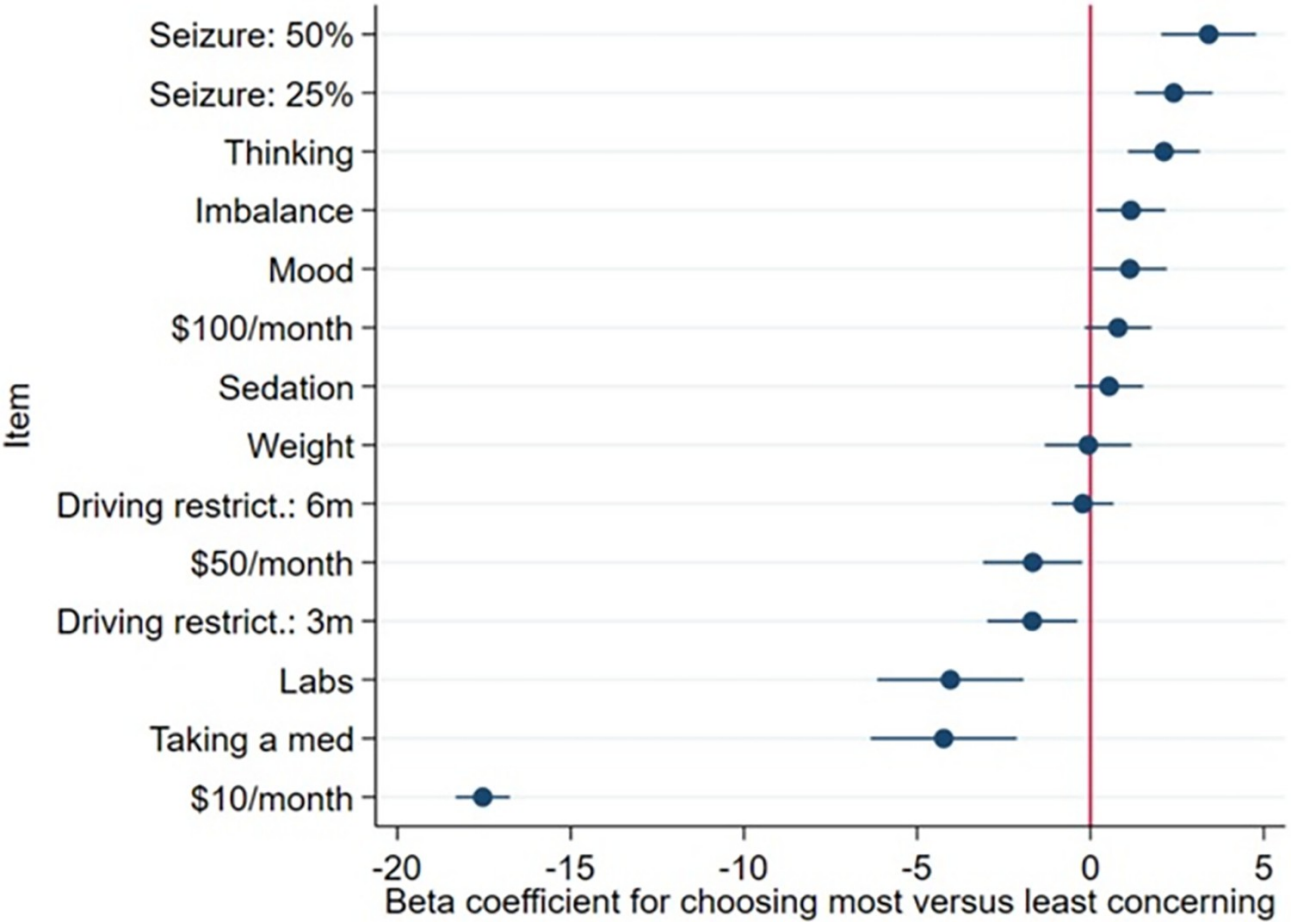
Coefficients and 95% confidence intervals for the patient multinomial logit of choosing each item as “most concerning” versus “least concerning”. The higher a coefficient, the greater the log(odds) was for choosing a given item as “most concerning” instead of “least concerning.” Thus, a 50% seizure risk was the most likely to be chosen as the most concerning item, whereas taking a medication was the least likely to be chosen as the most concerning. Coefficients are all in reference to a 10% seizure risk which is represented by the vertical red line.

Fig 3 displays coefficients from BWS patient responses on the log(odds) scale for each item, for choosing each item as the most important. We chose 10% seizure risk in the next year as the reference (the vertical line at 0), given its location in the middle of the range to anchor our comparisons at this lowest tier of seizure risk.

### ‘Inconsistent’ responses

Physicians made no inconsistent responses.

Patients did provide some inconsistent responses. For the VAS questions, 4 (13%) patients rated at least one higher seizure probability as being lower concern compared to a lower seizure probability. For example, one of those patients indicated that each increasing seizure risk monotonically corresponded to a lower degree of concern (VAS scores for 10%/25%/50% seizure risk: 40/28/25) suggesting they truly misunderstood the instructions. The other three patients provided orderings that were less clear (15/12/12, 30/21/31, and 10/51/50). For VAS questions, all patients rated $50/month as a higher concern than $10/month and thus there were no inconsistencies for cost.

Five out of the thirteen blocks had different levels of the same attribute which would allow testing for inconsistent BWS responses. Twelve (39%) patients made a total of thirteen inconsistent choices. These thirteen blocks represented 3% of the total 403 blocks we presented to patients (31 patients * 13 blocks/patient = 403 total blocks). The most common inconsistency was in block 4, where 7/31 (23%) patients ranked a 3-month driving restriction as more concerning than a 6-month driving restriction. In all seven instances, a 6-month driving restriction was chosen as the least concerning, either sedation or imbalance were chosen as the most concerning, and a 3-month driving restriction was never chosen as either most or least concerning. The next most common inconsistency was in block 1, where 5/31 (16%) ranked a 25% seizure risk as more concerning than a 50% seizure risk. In four of these five instances, the patient chose a 25% seizure risk as the most concerning. In one of these five instances, the patient chose a 50% seizure risk as the least concerning. The other two items were thinking difficulty and a 6-month driving restriction. The last inconsistency involved block 6, where one (3%) patient chose 10% seizure risk in the next year as the most concerning and did not mark a least concerning item (among sedation, laboratory monitoring, and 25% seizure risk).

Among 31 patients, 13 (42%) made an inconsistent response in either VAS or BWS questions. Among those 13, three made at least one inconsistent response in both VAS and BWS questions, nine made at least one inconsistent response in BWS questions only, and one made at least one inconsistent response in VAS questions only.

## Discussion

We report preliminary results developing and pilot testing a preference survey assessing factors influencing ASM treatment decisions in patients with well-controlled epilepsy. This pilot study was performed to optimize content and ensure comprehensibility prior to future full-scale data collection. Respondents generally agreed that the survey was understandable and complete, and they described areas for development prior to future full-scale data collection clarifying instructions and content.

One of the most interesting results of our work that will inform future questionnaire development was the presence of ‘inconsistent’ response patterns. While only 3% of BWS blocks had an inconsistent response, we still found that 39% of patients made at least one inconsistent response, and the most common pattern was to make an inconsistent response on BWS questions only. Inconsistencies particularly involved seizure probability and driving duration questions. For example, 7 patients chose a 6-month driving restriction as the least concerning and did not mark a 3-month driving restriction as either least or most concerning. This could have suggested they simply did not see the 3-month option (otherwise they would have logically marked that choice as the least concerning), or else did not understand the instructions. Seizure probabilities were the other main source of inconsistent responses, for example ranking a higher seizure risk as lower concern or vice versa. Patients with epilepsy have lower numeracy (the ability to comprehend and use numbers) than the general population [46]. This population is at high risk for cognitive impairment due to seizures, ASMs, and the underlying seizure etiology [47, 48], any of which could reduce numeracy. We initially attempted to mitigate this possibility by displaying seizure risk in terms of pictographs (showing 100 person icons, with the number of red person icons representing the number of people out of 100 that would have a seizure under a given percentage) informed by optimal risk communication literature [49]. However, both physicians and patients found these charts cluttering and more confusing that displaying percentages only. In the future, we may consider showing fractions (“1 in 10”) instead of percentages (“10%” also informed by risk communication literature [49] which could be clearer, and also consider only listing a single level of driving restrictions relevant to the patient’s US state to reduce as much confusion as possible. One could have hypothesized that errors would increase by the end of the survey due to respondent fatigue, but all BWS errors occurred in the first half of the BWS questions suggesting against this. While we did include a static ‘example’ BWS response to show respondents what a correctly filled out item would look like, in the future we may include an interactive BWS question requiring that respondents fill out an example question correctly before proceeding. Other strategies may be either collapsing ‘having a seizure’ into a single item without distinguishing between different risk percentages which may have been confusing to patients, or else to force the survey design to allow only a single seizure risk level per block, i.e., displaying an ‘error message’ warning the respondent that they may wish to revise their choice. We intentionally allowed more than one seizure risk level per block though in this pilot study to evaluate the degree to which patients were understanding the task, and because our a priori goal was to evaluate what seizure risk patients believe outweighs each downside of treatment. Interestingly, inconsistencies were not seen with cost items–it is plausible that people have a much more intuitive sense of dollars compared with risk percentages, thus our future work may modify driving duration or seizure risk items but there is no need to modify the cost items. While evaluating for such ‘inconsistent’ responses represented a key piece of this pilot testing, we will also evaluate for ‘inconsistent’ responses in our future larger-scale study and compare responses with randomly generated choices to identify outliers. We will execute sensitivity analyses both including and excluding such inconsistent responses to evaluate the robustness of conclusions.

It was encouraging that results tended to align between techniques—cost, lab monitoring, and the inconvenience of pill-taking were rated as the lowest concerns, whereas cognitive side effects and the highest tier risk of seizure were rated as the highest concerns, for both VAS and BWS questions. This supports convergent validity between techniques. Our preliminary results also suggested that numerous items had wide variation, particularly for the importance of driving restrictions, and greater variation appeared to exist for VAS than BWS questions. This argues for collecting baseline data from future respondents that may influence the importance of driving, such as whether patients work, have dependents, availability of public transportation, and cognitive ability, to further individualize results across clinically important subgroups.

Patient preference studies have immediate clinical implications, because understanding what range of seizure risk outweighs which side effects and ASM-related inconveniences provides key information towards understanding which patients may benefit from continued treatment. Thus, guideline developers and policy makers need evidence on patient preferences to optimally inform patient-centered care [16]. Even if in our small sample of physicians appeared qualitatively similar to patient responses, literature supports that physicians frequently misestimate patient preferences [50–54]. Prior work in larger samples has suggested that neurologists rank seizure reduction significantly more important and side effect reduction significantly less important compared with what patients actually value [34]. Across common medical decisions providers do not ask about patient preferences in up to two-thirds of encounters before recommending interventions [55], and only one-third of patients with epilepsy seizure-free for >5 years have ever discussed the possibility of discontinuation with their physician [56]. This is particularly problematic because patients frequently do not voice their true concerns during time-limited office visits without a structured process for preference measurement [53]. Thus, research developing structured patient preference elicitation exercises are critical to inform the medical community about patient priorities, both in epilepsy and across other medical conditions involving preference-sensitive treatment decisions. BWS results may also inform guideline development, as incorporating systematic studies of patient preferences may help policymakers understand how patients with different values may reach different healthcare decisions [16].

Our work has several limitations. It was performed at a single academic center with a predominantly Caucasian patient population and thus results may not generalize well to non-academic centers or other populations. Future data collection could also be expanded to patients with poorly controlled epilepsy, and cases managed by non-specialists. It is also not possible to include all items that may possibly influence decision making within a single survey. In this pilot we did not include ASM-related birth defects which would be relevant to women of childbearing age. Though, no respondent identified this was an important omission, and the only noted possible omission was that two respondents noted ASM-related psychosis could be an item to consider in our future larger-scale survey. We may still provide an open-ended question in our future larger-scale survey for respondents to share any important considerations still not captured in our final instrument. This pilot study was not intended to be powered to detect between-patient differences or latent classes of patient preferences which can help clarify if a decision is preference-sensitive, or to formally compare patient versus physician responses. Our future work may more formally compare physician versus patient responses and will be planned to have sufficient power to explore patient heterogeneity. For example, given approximately half of participants’ medical charts did not inform whether the patient was driving, this pilot suggested we should embed this as a future survey question. Finally, the decision to continue or discontinue a medication is a bundled choice profile, e.g., discontinuing implies both a higher risk of seizures and thus a higher risk of driving restrictions and a lower risk of side effects. Thus, splitting each attribute into separate items does not necessarily mimic true clinical practice. Choices regarding whether to modify or discontinue ASMs should be made within a patient’s unique holistic context ideally by clinicians experienced in caring for patients with epilepsy. Nonetheless, our survey was designed to disentangle the relative importance of each attribute to inform factors influencing the decision-making process.

## Conclusions

We described the development and pilot testing of a BWS survey intended to examine patient preferences in well-controlled epilepsy. Recruitment rate and time to complete questions were reasonable, respondents mostly agreed the questions were clear and assessed preferences well, and respondents provided feedback to improve the survey prior to full-scale data collection. ‘Inconsistent’ responses will inform survey refinement efforts. Cost, lab monitoring, and the inconvenience of pill-taking were rated as the lowest concerns, whereas cognitive side effects and the highest tier risk of seizure were rated as the highest concerns. Preference elicitation exercises such as this may both inform clinicians about how strongly patients value certain outcomes over others when deciding upon treatment options with pros and cons and could be useful in the future by informing policy-relevant patient-centered guideline development.

## Supporting information

S1 Checklist STROBE 2007 (v4) statement—checklist of items that should be included in reports of *cross-sectional studies*. (DOC)

S1 Appendix Physician survey. (PDF)

S2 Appendix Patient survey. (PDF)
