# Correction: Best-worst scaling preferences among patients with well-controlled epilepsy: Pilot results

**DOI:** 10.1371/journal.pone.0322210

**Published:** 2025-04-04

**Authors:** Samuel W. Terman, Hélène E. Aschmann, David W. Hutton, James F. Burke

In the Funding statement, the grant number from the funder Early Postdoc.Mobility Fellowship by the Swiss National Science Foundation is not indicated. The grant number is: 191414.
