# Supplementary material for: Best-worst scaling preferences among patients with well-controlled epilepsy: Pilot results
Source: PLoS One. 2023 Mar 3;18(3):e0282658. doi: 10.1371/journal.pone.0282658 (PMC9983827; doi:10.1371/journal.pone.0282658)
Supplement: S1 Appendix — (PDF) [file pone.0282658.s002.pdf]

Page Submit: *0 seconds*
Click Count: *0 clicks*

To familiarize you with our survey, here are the types of scenarios we will ask you to consider. *We realize that some of this might not apply to your particular situation, and sometimes choices will appear that may not go together in real life. That is ok - your answers will help doctors understand how patients think about different parts of their epilepsy and medications.*

Seizure risk: Imagine you had one of the following risks for having at least one seizure in the next year. Options will range between 10% (on the left) to 50% (on the right).

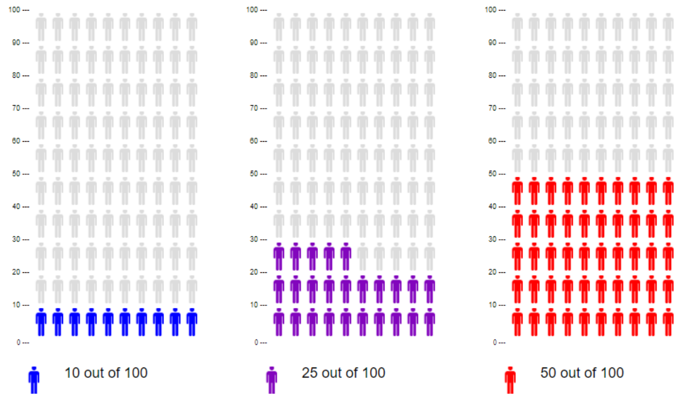

Driving restriction: Imagine you were not legally allowed to drive for the next 6 months.

Medication-related side effects: Imagine you had side effects due to medications that were mild, but enough to notice them in your day to day function. There may include mood changes (such as irritability or sadness), thinking/memory difficulty (such as coming up with words or storing new information), sedation (extra drowsiness), weight gain (a couple pounds), or imbalance/dizziness (unsteadiness when walking).

Laboratory monitoring: Imagine you had to have your blood drawn once every 6 months to make sure you blood counts were in a safe range.

Taking a seizure medication: Imagine you had to take a medication to control seizures twice daily.

Cost: Imagine you had a given out of pocket expense each month, such as $10 or $50.

Let's begin the questions.

Please rate each of the following from not at all concerning (0) to extremely concerning (100). Click within the bar under each item to mark your choice.

	Not at all concerning		Somewhat concerning		Extremely concerning		
	0	20	40	60	80	100	
Seizure risk in the next year: 10%							0
Seizure risk in the next year: 25%							0
Seizure risk in the next year: 50%							0
Driving 6 month restriction							0
Medication-related mood changes							0
Medication-related thinking/memory difficulty							0
Medication-related sedation							0
Medication-related weight gain							0
Medication-related imbalance/dizziness							0
Laboratory monitoring every 6 months							0
Taking a seizure medication twice daily							0

	Not at all concerning		Somewhat concerning		Extremely concerning	
	0	20	40	60	80	100
$10/month seizure medication cost						0
$50/month seizure medication cost						0

Part 2: Best-worst scaling

These page timer metrics will not be displayed to the recipient.
First Click: *0 seconds*
Last Click: *0 seconds*
Page Submit: *0 seconds*
Click Count: *0 clicks*

Now, here is another type of question. This may be more challenging.

For each group of 4 items below, mark two total choices: one in the left column for the single least concerning item in the group, and another one in the right column for the single most concerning item in the group. Repeat this process for every group of 4 items you see below.

Here's an example to help you understand the instructions. Can you practice marking the most and least concerning items from this list? Just make 2 marks: ONE on the left (which item LEAST concerns you?) and ONE on the right (which item MOST concerns you?).

Least concerning		Most concerning
○	The most concerning item (choose the button to the right of this)	○
○	Another item	○
○	The least concerning item (choose the button to the left of this)	○

Least concerning		Most concerning
○	Another item	○

Now, let's begin the actual questions. There are no right or wrong answers - only what matters most and least to you.

Least concerning		Most concerning
○	Medication-related thinking/memory difficulty	○
○	Seizure risk in the next year: 50%	○
○	Driving 6 month restriction	○
○	Seizure risk in the next year: 25%	○

Least concerning		Most concerning
○	Driving 6 month restriction	○
○	Taking a seizure medication twice daily	○
○	Laboratory monitoring every 6 months	○
○	$50/month seizure medication cost	○

Least concerning		Most concerning
○	Medication-related mood changes	○
○	Driving 6 month restriction	○
○	$10/month seizure medication cost	○
○	Seizure risk in the next year: 10%	○

Least concerning		Most concerning
○	Medication-related weight gain	○

Least concerning		Most concerning
○	Medication-related sedation	○
○	Medication-related imbalance/dizziness	○
○	Driving 6 month restriction	○

Least concerning		Most concerning
○	Taking a seizure medication twice daily	○
○	Seizure risk in the next year: 10%	○
○	Medication-related thinking/memory difficulty	○
○	Medication-related weight gain	○

Least concerning		Most concerning
○	Medication-related sedation	○
○	Seizure risk in the next year: 25%	○
○	Seizure risk in the next year: 10%	○
○	Laboratory monitoring every 6 months	○

Least concerning		Most concerning
○	Seizure risk in the next year: 50%	○
○	Medication-related mood changes	○
○	Taking a seizure medication twice daily	○
○	Medication-related sedation	○

Least concerning Most concerning

Least concerning		Most concerning
○	Seizure risk in the next year: 25%	○
○	Medication-related weight gain	○
○	$50/month seizure medication cost	○
○	Medication-related mood changes	○

Least concerning		Most concerning
○	$50/month seizure medication cost	○
○	Medication-related thinking/memory difficulty	○
○	Medication-related sedation	○
○	$10/month seizure medication cost	○

Least concerning		Most concerning
○	$10/month seizure medication cost	○
○	Medication-related imbalance/dizziness	○
○	Seizure risk in the next year: 25%	○
○	Taking a seizure medication twice daily	○

Least concerning		Most concerning
○	Medication-related imbalance/dizziness	○
○	Laboratory monitoring every 6 months	○
○	Medication-related mood changes	○
○	Medication-related thinking/memory difficulty	○

Least concerning		Most concerning
◯	Laboratory monitoring every 6 months	◯
◯	$10/month seizure medication cost	◯
◯	Medication-related weight gain	◯
◯	Seizure risk in the next year: 50%	◯

Least concerning		Most concerning
◯	Seizure risk in the next year: 10%	◯
◯	$50/month seizure medication cost	◯
◯	Seizure risk in the next year: 50%	◯
◯	Medication-related imbalance/dizziness	◯

Part 3: Feedback

Thank you for completing this survey.

This is the end of the portion that respondents will see. We have a few followup questions regarding your experience to improve our survey, prior to full data collection.

The survey duration was:

- ◯ Much too short
- ◯ A bit too short
- ◯ Just right
- ◯ A bit too long
- ◯ Much too long

For the best-worst questions:

	Strongly agree	Somewhat agree	Neither agree nor disagree	Somewhat disagree	Strongly disagree
Instructions were clear.	○	○	○	○	○
Interface was easy to use.	○	○	○	○	○
Assessed my preferences related to AEDs well.	○	○	○	○	○
Easy to answer.	○	○	○	○	○

For the rating questions:

	Strongly agree	Somewhat agree	Neither agree nor disagree	Somewhat disagree	Strongly disagree
Instructions were clear.	○	○	○	○	○
Interface was easy to use.	○	○	○	○	○
Assessed my preferences related to AEDs well.	○	○	○	○	○
Easy to answer.	○	○	○	○	○

That is the end of our survey. Thank you for your participation.

Powered by Qualtrics
