# Supplementary material for: Best-worst scaling preferences among patients with well-controlled epilepsy: Pilot results
Source: PLoS One. 2023 Mar 3;18(3):e0282658. doi: 10.1371/journal.pone.0282658 (PMC9983827; doi:10.1371/journal.pone.0282658)
Supplement: S2 Appendix — (PDF) [file pone.0282658.s003.pdf]

Introduction

This page is just an introduction. The next page will start the actual questions.

First, we will ask you to 'rate' how concerning different things would be if they were hypothetically true, on a scale of 0-100 . For example, 'what if the chance of having a seizure in the next year was 10%?' Or, 'what if it was 25%'? Or, 'what if you had this or that particular medication side effect?' There will be similar questions about driving restrictions, lab monitoring, and cost.

Second, we will show you a smaller number of those items at a time, and ask you to pick which would be most concerning and which would be least concerning. It will make more sense when you see the actual questions.

Third, we will ask you for any feedback you may have on how to improve this survey for future participants.

Rating questions

Here is the first set of questions.

Click and drag the slider bar for each item below between 0 (left, not at all concerned, i.e. perfect health) and 100 (right, extremely concerned, i.e. death), to indicate how concerned you would be if each item were hypothetically true. Do this for each, even if you think the answer should be 0 and even if something may not apply to you currently.

How concerned would you be if you had or your doctor said you would have...

Not at all concerned	Somewhat concerned	Extremely concerned

0 10 20 30 40 50 60 70 80 90 100

	Not at all concerned				Somewhat concerned				Extremely concerned	
	0	10	20	30	40	50	60	70	80	90	100

Seizure risk in the next year: 10%

Seizure risk in the next year: 25%

Seizure risk in the next year: 50%

Driving 6 month restriction

Medication-related mood changes

Medication-related thinking/memory difficulty

Medication-related sedation

Medication-related weight gain

Medication-related imbalance/dizziness

Laboratory monitoring every 6 months

Taking a seizure medication twice daily

$10/month seizure medication cost

$50/month seizure medication cost

Best-worst scaling questions

Here is the second type of question.

For each group of 4 items, just mark 2:
1) One in the **left** column - the item which would be **least concerning** to you.
2) One in the **right** column - the item which would be **most concerning** to you.

The items will get repeatedly shuffled, on purpose, so each comparison is slightly different each time.

Here is an example picture just to show what a response would look like. Of these 4 items, say you found Item 1 the least concerning, and Item 2 the most concerning:

Least concerning		Most concerning
●	Item 1	○
○	Item 2	●
○	Item 3	○
○	Item 4	○

Now, let's begin the actual questions.

1/13:

Least concerning		Most concerning
○	Medication-related thinking/memory difficulty	○
○	Seizure risk in the next year: 50%	○
○	Driving 6 month restriction	○
○	Seizure risk in the next year: 25%	○

2/13:

Least concerning		Most concerning
○	Driving 6 month restriction	○
○	Taking a seizure medication twice daily	○
○	Laboratory monitoring every 6 months	○
○	$100/month seizure medication cost	○

3/13:

Least concerning		Most concerning
○	Medication-related mood changes	○
○	Driving 6 month restriction	○
○	$10/month seizure medication cost	○
○	Seizure risk in the next year: 10%	○

4/13:

Least concerning		Most concerning
○	Driving 3 month restriction	○
○	Medication-related sedation	○
○	Medication-related imbalance/dizziness	○
○	Driving 6 month restriction	○

5/13:

Least concerning		Most concerning
○	Taking a seizure medication twice daily	○
○	Seizure risk in the next year: 10%	○
○	Medication-related thinking/memory difficulty	○
○	Driving 3 month restriction	○

6/13:

Least concerning		Most concerning
○	Medication-related sedation	○
○	Seizure risk in the next year: 25%	○
○	Seizure risk in the next year: 10%	○
○	Laboratory monitoring every 6 months	○

7/13:

Least concerning		Most concerning
○	Seizure risk in the next year: 50%	○
○	Medication-related mood changes	○
○	Taking a seizure medication twice daily	○
○	Medication-related sedation	○

8/13:

Least concerning		Most concerning
○	Seizure risk in the next year: 25%	○
○	Medication-related weight gain	○
○	$50/month seizure medication cost	○
○	Medication-related mood changes	○

9/13:

Least concerning		Most concerning
○	$100/month seizure medication cost	○
○	Medication-related thinking/memory difficulty	○
○	Medication-related sedation	○
○	$10/month seizure medication cost	○

10/13:

Least concerning		Most concerning
○	$10/month seizure medication cost	○
○	Medication-related imbalance/dizziness	○
○	Seizure risk in the next year: 25%	○
○	Taking a seizure medication twice daily	○

11/13:

Least concerning		Most concerning
○	Medication-related imbalance/dizziness	○
○	Laboratory monitoring every 6 months	○
○	Medication-related mood changes	○
○	Medication-related thinking/memory difficulty	○

12/13:

Least concerning		Most concerning
○	Laboratory monitoring every 6 months	○
○	$10/month seizure medication cost	○
○	Medication-related weight gain	○
○	Seizure risk in the next year: 50%	○

13/13:

Least concerning		Most concerning
○	Seizure risk in the next year: 10%	○
○	$100/month seizure medication cost	○
○	Seizure risk in the next year: 50%	○
○	Medication-related imbalance/dizziness	○

Feedback

We will be done in just a moment. Please provide feedback about to help us improve our survey in the future.

The survey duration was:

- ◯ Much too short
- ◯ A bit too short
- ◯ Just right
- ◯ A bit too long
- ◯ Much too long

For the rating questions (the first part):

	Strongly agree	Somewhat agree	Neither agree nor disagree	Somewhat disagree	Strongly disagree
Instructions were clear.	◯	◯	◯	◯	◯
Interface was easy.	◯	◯	◯	◯	◯
Assessed my preferences related to seizure medications well.	◯	◯	◯	◯	◯
Easy to answer.	◯	◯	◯	◯	◯

For the 'most/least concerning' questions (the second part):

	Strongly agree	Somewhat agree	Neither agree nor disagree	Somewhat disagree	Strongly disagree
Instructions were clear.	◯	◯	◯	◯	◯

	Strongly agree	Somewhat agree	Neither agree nor disagree	Somewhat disagree	Strongly disagree
Interface was easy.	○	○	○	○	○
Assessed my preferences related to seizure medications well.	○	○	○	○	○
Easy to answer.	○	○	○	○	○

Anything else you'd like to share?

Powered by Qualtrics
